# Cca-miR398 increases copper sulfate stress sensitivity via the regulation of* CSD* mRNA transcription levels in transgenic* Arabidopsis thaliana*

**DOI:** 10.7717/peerj.9105

**Published:** 2020-05-26

**Authors:** Zhichao Sun, Lilu Shu, Wei Zhang, Zhengjia Wang

**Affiliations:** State Key Laboratory of Subtropical Silviculture, Zhejiang A&F University, Hangzhou, Zhejiang, China

**Keywords:** Arabidopsis, cca-miR398, *CSD*, Copper sulfate, Hickory, qRT-PCR

## Abstract

MicroRNAs play crucial roles during the process of plant development under stress conditions. Copper is an essential micronutrient for most organisms and serves as an important redox-active cofactor for various functional proteins. In the present study, we investigated the effects of copper sulfate stress on hickory (*Carya cathayensis*) root development. We identified that hickory cca-miR398 was related to copper sulfate stress response, targeting Copper/Zinc superoxide dismutases (cytosolic (*CSD1*) and chloroplastic (*CSD2*)) and a 5b subunit of mitochondrial cytochrome C oxidase (*COX5b.1*) that are linked directly to stress regulatory networks. The sequence of hickory cca-miR398 is highly similar to that of Arabidopsis miR398b and miR398c, regardless of one nucleotide variation. Therefore, target genes of cca-miR398 were investigated by using 5′-Rapid-amplification of cDNA ends. An overexpression of cca-miR398 in Arabidopsis caused a reduction not only in root length and cotyledon greening, but also in the *CSD1*, *CSD2,* and *CSD3* transcription levels. These reductions had greater significance in transgenic Arabidopsis than in wild-type Arabidopsis under copper sulfate stress. The level of physiological indicators also changed in transgenic Arabidopsis. In addition, the expressions of copper-responsive microRNAs, such as miR397 and miR408, were affected by the copper sulfate stress. These results showed that *CSD* possesses the ability to enhance copper sulfate stress response in both transgenic Arabidopsis and hickory roots by increasing the production of superoxide dismutase. Our results also demonstrated that cca-miR398 weakens hickory tolerance to copper sulfate by regulating *CSD* targets.

## Introduction

Copper (Cu), which serves as an essential redox-active cofactor, participates in numerous fundamental processes of plant biology. Despite it being essential to agriculture, an excess of copper causes various destructive effects on plant growth, such as chlorosis of vegetative tissue and the abnormal developments of roots, flowers, and seeds (Guan et al., 2017; Nie et al., 2015; Zhang et al., 2017). To survive and propagate, plants have developed diverse mechanisms to adapt to stress, such as accumulating different kinds of osmolytes (He et al., 2018) that often include proline, glycine, trigonelline, and antioxidant enzymes. To meet the survival needs, plants also adopted strategies to balance osmotic pressure and regulate cell membrane stability (Hossain et al., 2015; Jajic, Sarna & Strzalka, 2015).

Superoxide dismutase (SOD) enzymes are widely distributed in a variety of organisms, scavenging superoxide radicals while releasing molecular oxygen and hydrogen peroxide. SOD enzymes also crucially remove free radicals generated in the mitochondrial respiratory pathway under stress (Farhangi-Abriz & Torabian, 2017; Li et al., 2014).

MicroRNA (miRNA) is a small non-coding RNA with 20–24 nucleotides. In plants, miRNAs regulate various developmental processes and stress adaptations by inhibiting the expression of a target gene at both transcriptional and post-transcriptional levels. They have been reported in many species and found to be involved in regulation, metabolism, growth, development, and other physiological processes in plants. For example, the miR398 of *Taraxacum officinale* can be an indicator of heavy metals (e.g., aluminium and cadmium) from air pollution (Gómez-Arroyo et al., 2018). Specifically, miR398 targets Cu/Zn SODs (CSDs) and a 5b subunit of mitochondrial cytochrome C oxidase (*COX5b.1*), which connect directly to stress regulatory networks. The complementarity between miR398 and its three gene targets (*CSD1*, *CSD2* and *COX5b.1*) is conserved among Arabidopsis, rice, and pine (Araki et al., 2018; Kundu et al., 2017). In CSD2 mRNA, miR398 anchors its complementarity site at the coding region (Araki et al., 2018; Lu, Guan & Zhu, 2013). However, miR398 targets the 5′-UTR of *CSD1* and *COX5b.1* transcripts (Araki et al., 2018). MiR398 silences mRNA molecules by cleaving the mRNA in two (*CSD2*) and inhibits protein translation with completely complementary sequences (*CSD1* and *COX5b.1*; Araki et al., 2018; Lu, Guan & Zhu, 2013). Previous studies have indicated that CSD1 and CSD2 mRNA levels increased as miR398 levels decreased when Arabidopsis seedlings were exposed to high concentrations of Cu^2+^ or Fe^3+^ (Abdel-Ghany & Pilon, 2008; Sunkar, Kapoor & Zhu, 2006). Although the functions of miR398 in Arabidopsis under abiotic stress conditions (e.g., drought and high salinity) are known (Araki et al., 2018; Sunkar, Kapoor & Zhu, 2006), it remains unclear if miR398 has the distinct function of mediating stress responses in hickory.

In this study, we provide experimental evidence that the application of copper sulfate has a significant effect on hickory root development. By locating miR398 and *CSDs* in a hickory transcriptome database, we obtained homologous hickory miR398 named “cca-miR398”. Our sequence analysis showed that, apart from a single nucleotide difference, cca-miR398 was highly similar to miR398b and c sequences of Arabidopsis. The quantitative real-time PCR (qRT-PCR) analysis of the expression profiles of cca-miR398 and *CSDs* has suggested that these genes were involved in stress tolerance. To characterize cca-miR398 further, we constructed transgenic Arabidopsis that constitutively over-expresses cca-miR398. The transgenic Arabidopsis turned out have lower stress tolerance and shorter roots than wild-type (WT) Arabidopsis. Additionally, the levels of stress-related physiological indicators were altered and *CSD* expression levels (e.g., *CSD3*) decreased.

## Material and Methods

### Copper sulfate stress assays in hickory

Hickory seeds were germinated indoors under artificial climate conditions (25 °C, 100 µmol photons m^−2^s^−1^, 60% relative humidity, 16/8 day/night cycles). The 30-day-old hickory roots were subjected to the following stress treatment conditions: 0, 100 and 200 mM copper sulfate. 10 days following treatment, the lengths of 30 hickory roots from each treatment group were surveyed and photographed. Roots were then reaped 10 days after treatment, and the samples were stored immediately at −80 °C for RNA isolation. The expression patterns of cca-miR398 and *CSDs* (Table S1) were then examined by qRT-PCR.

### Cca-miR398 constructs and the generation of transgenic Arabidopsis

The precursor of cca-miR398 was PCR-amplified from the hickory roots. The cca-miR398-overexpression vector was made based on the vector pMD19-simple of T-Vector pMD™ 19 kit (TaKaRa, China). A fragment of 400 bp mRNA flanking the miRNA sequence (Table S2), including the fold-back motif, was inserted into the vector. The precursor of cca-miR398 was ligated into pCAMBIA13011, which was pre-digested by *XbaI* and *KpnI*. The resulting construct (containing pCAMBIA13011 and cca-miR398 precursor) was ligated with T4 DNA ligase, transferred into an agrobacterium tumefaciens EHA105 strain, and then used to infect an Arabidopsis ecotype “Columbia” (Col-0) via the floral dipping method (Mara, Grigorova & Liu, 2010). Transgenic lines were subsequently selected with hygromycin B and confirmed with PCR amplification.

### Copper sulfate stress assays in transgenic and WT Arabidopsis

Primary Arabidopsis transformants were selected on Murashige-Skoog (MS) medium (Murashige & Skoog, 1962) supplemented with 50 mg L^−1^ hygromycin B. For seed germination root length determination, the seeds were sterilized and placed on three varieties of MS agar medium supplemented with different concentrations of copper sulfate (40, 80, and 160 mM). The seeds were then germinated under artificial climate room conditions (25 °C, 100 µmol photons m^−2^s^−1^, 60% relative humidity, 16/8 day/night cycles). The germination rate was counted, and pictures were taken on the seventh day. Five-day-old seedlings were separately transferred to medium supplemented with copper sulfate (40, 80, and 160 mM). After eight days of growth, the root lengths were surveyed and photographed.

For copper sulfate tolerance assessment, transgenic seeds and WT seeds were sown in pots for four weeks. The seeds were grown under artificial climate room conditions (25 °C, 100 µmol photons m^−2^s^−1^, 60% relative humidity, 16/8 day/night cycles). During the fourth week, plants were irrigated with various concentrations copper sulfate (0, 500, 1,000, 1,500, and 2,000 mM). After 15 days of treatment, related physiological indicators were detected and leaves were respectively harvested from each treatment group before being stored at −80 °C until RNA isolation. Expression patterns of candidate genes (Table S1) were then examined by qRT-PCR after RNA isolation and first-strand cDNA synthesis.

### Expression analysis by qRT-PCR

RNA samples extracted from Arabidopsis leaves and hickory roots were subjected to qRT-PCR. The transcription of miRNA of the Arabidopsis and hickory was completed using a Mir-XTM miRNA First-Stand Synthesis Kit (TaKaRa, China). The reactions were performed with a CFX96 Real-time PCR Detection System (BioRad, U.S.A) using a 96-well format. The qRT-PCR process consisted of one cycle of 30 s at 95 °C followed by 40 cycles of 95 °C for 5 s and 60 °C for 34 s. All reactions included nine replicates (three biological triplicates and three technical triplicates). Data were analyzed with CFX96 system software (BioRad, U.S.A), and the relative gene expression levels in various tissues were calculated based on the 2^−ΔΔCT^ method (Livak & Schmittgen, 2001). The actin and 5.8S rRNA were used as a reference gene for *CSD* genes and miRNA, respectively. The qRT-PCR primers are listed in Table S3.

### Measurements of physiological indicators

Physiological indicators refer to the activities of SOD, peroxidase (POD), and catalase (CAT) in Arabidopsis leaves. These activities were determined using respective SOD, POD, and CAT assay kits (A001-2, Nanjing, China). An IMAGING-PAM chlorophyll fluorometer (Heinz Walz GmbH, Effeltrich, Germany) was used to record the fluorescence intensity readout (Shao et al., 2008). A portion at each leaf center with a 1-cm diameter was selected to provide the measurements. The means of chlorophyll fluorescence parameters, including fluorescence images of the maximal Y(II) quantum yield (Fv/Fm), an index of the photochemical efficiency of effective PSII quantum yield system (PSII), the electron transport rate (ETR), non-photochemical quenching (NPQ), and the coefficient of photochemical quenching (qP), were calculated. Fv/Fm fluorescence images were derived simultaneously from the IMAGING-PAM software.

### Statistical analysis

Statistical analyses were performed by using SPSS version 13.0. Differences were analyzed based on a one-way ANOVA model followed by Tukey’s range test.

## Results

### Morphological effects in hickory roots under copper sulfate stress

**Figure 1:**
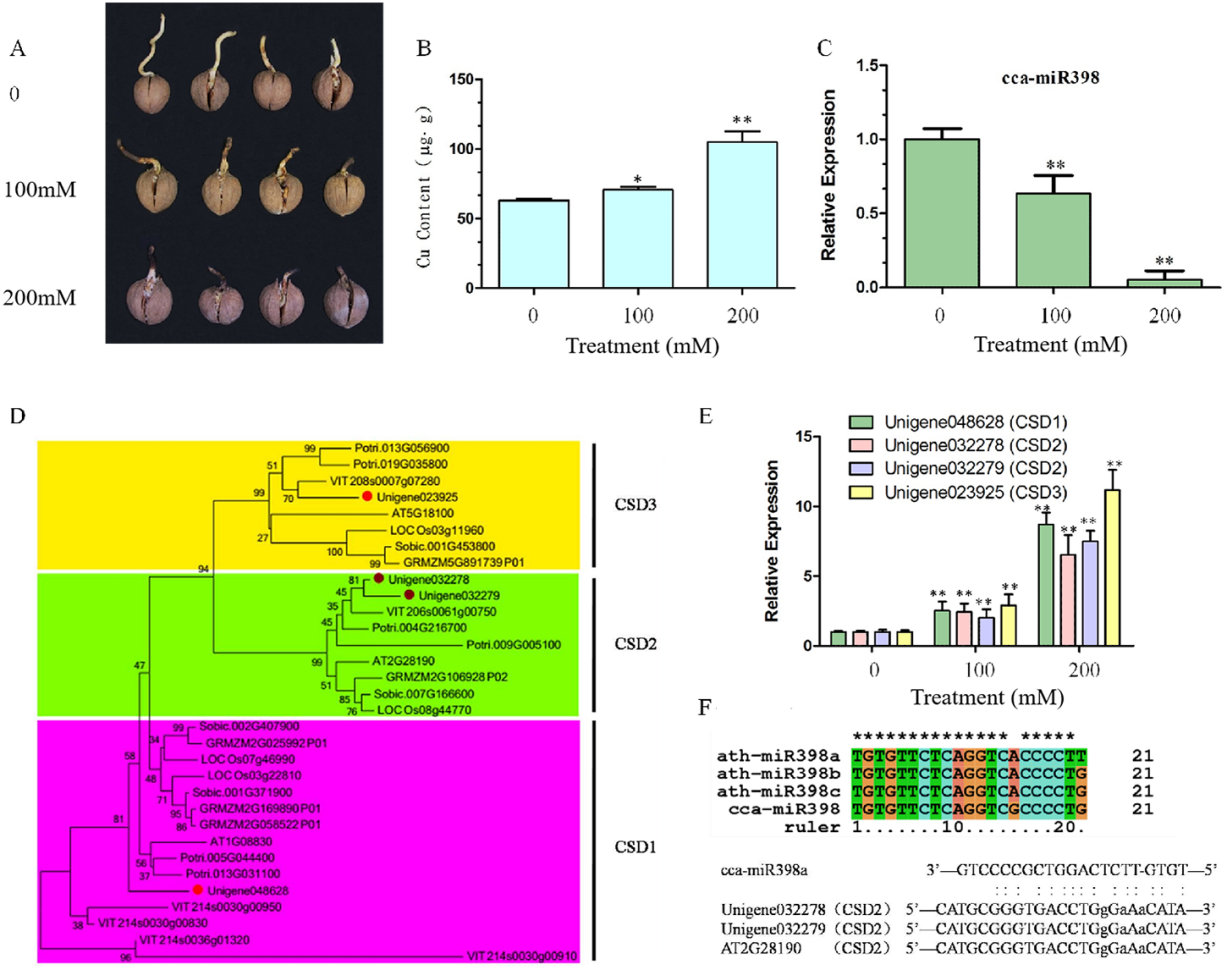
Copper sulfate stress assays and expression of related genes in hickory roots. (A) Root growth of hickory seed exposed to 0, 100, and 200 mM copper sulfate. (B) Copper element accumulation in hickory roots exposed to copper stress for seven days. (C) Relative expression of cca-miR398 in hickory roots exposed to 0, 100, and 200 mM copper sulfate. (D) Phylogenetic tree analysis of the *CSD* nucleotide gene sequences from different plant species. Gene sequences and their accession numbers were selected from the Phytozome genome portal and depicted in the tree. The hickory *CSD* genes are marked with red filled circles. The phylogenetic tree was constructed with the MEGA 6.02 program using the Neighbor-Joining method with a bootstrap value of 1,000. The numbers at the nodes indicate the bootstrap values. (E) Expression of *CSDs* in hickory exposed to 0, 100, and 200 mM copper sulfate. (F) Sequence alignment of the mature cca-miR398 with Arabidopsis miR398 family members, alignment of cca-miR398, and target *CSD2s*. Data are means ± SE. Asterisks indicate a significant difference between the treatments and the control (*P < 0.05; **P < 0.01).

After 10 days of copper sulfate stress treatment, hickory root development was observed to be significantly different among the three concentrations. Root growth was reduced seriously by increasing the concentration of copper sulfate (Fig. 1A), and more copper sulfate was absorbed into the hickory root in the 200 mM group than in the 100 mM group (Fig. 1B). Relative expression of cca-miR398a significantly decreased (*P* < 0.01) with increased concentrations of copper sulfate (Fig. 1C).

### Sequence comparison and gene expression of CSDs in hickory roots

*CSD* genes of Arabidopsis, poplar, grape, rice, sorghum, and corn were downloaded from Phytozome v 10.3 (http://phytozome.jgi.doe.gov/pz/portal.html). A phylogenetic comparison of their sequences with hickory *CSDs* is shown in Fig. 1D. Hickory *CSDs* were identified using the database with respect to the corresponding class (*CSD1*, *CSD2*, and *CSD3*), and were distributed into three subfamilies, as shown in Fig. 1D. The cluster analyses indicated that they were derived from a common ancestor, although *CSD2* and *CSD3* appeared to be more closely related, both evolutionarily and structurally. Thus, three *CSD*-like genes (Unigene048628, Unigene032279, and Unigene032278) were predicted to be hickory orthologues of the *CSD* family in Arabidopsis (Fig. 1D). The qRT-PCR analysis was used to investigate whether *CSD* genes were involved in the regulation of abiotic stress in hickory roots. Transcript levels of *CSDs* in the roots significantly increased (*P* < 0.01) under the copper sulfate treatments and the expression patterns of *CSDs* were similar to each other (Fig. 1E).

### Sequence comparison of cca-miR398 with Arabidopsis miR398 family members

To analyze the functions of cca-miR398, a sequence comparison of miR398 family members from hickory and Arabidopsis was performed. As shown in Fig. 1F, the mature sequence of cca-miR398 had only one nucleotide variation (at position 15) compared to miR398b and c sequences of Arabidopsis. Variation with two nucleotides was observed (at positions 15 and 21) between cca-miR398 and miR398a (Fig. 1F).

In an Arabidopsis model plant, miR398 has been shown to target two *CSD* genes, *CSD1* and *CSD2* (Li et al., 2017; Mermod et al., 2019). In Arabidopsis *CSD2* mRNA, a complementarity site of miR398 is in the coding sequence (Li et al., 2017; Mermod et al., 2019), which is identical in hickory *CSD2* (Fig. 1F). In Arabidopsis *CSD1* mRNA, the complementarity site of miR398 is in the 5′-UTR and contains an intron interruption (Li et al., 2017; Mermod et al., 2019).

The sequence of 5′-UTRs in *CSD1* and *CSD3* are still not available in current hickory database, making a detailed comparison of the 5′-UTR sequences impossible. However, based on the high level of sequence similarity in coding regions, hickory *CSD1* and *CSD3* may also contain the miR398 complementarity site in their 5′-UTRs.

### Influences of copper sulfate on phenotypes of WT and cca-miR398 overexpressed transgenic Arabidopsis

**Figure 2:**
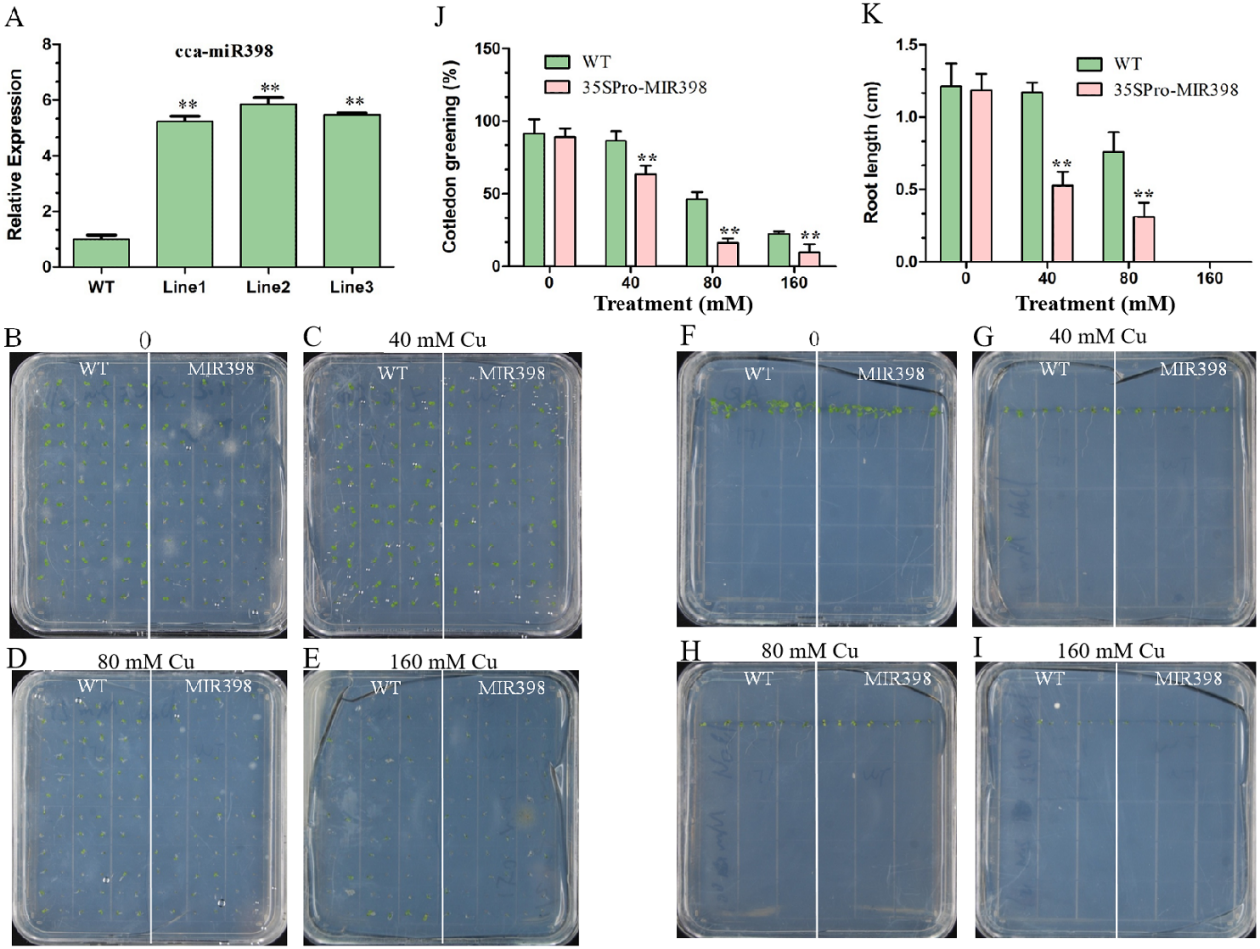
Cotyledon greening and roots growth responses of transgenic Arabidopsis to copper sulfate stress. (A) The cca-miR398 abundance was analyzed in WT and transgenic Arabidopsis. (B–I) Seeds of the WT and transgenic plants were germinated on plates with and without copper sulfate. Cotyledon greening and root lengths of WT and transgenic plants exposed copper sulfate. (J) The data of cotyledon greening (%) and (K) root lengths (cm) of WT and transgenic Arabidopsis. Data are means ± SE. Asterisks indicate a significant difference between the transgenic lines and the WT (**P < 0.01).

The expression of cca-miR398 was up-regulated (*P* < 0.01) in transgenic Arabidopsis (Fig. 2A). Seed germination of transgenic Arabidopsis was more sensitive than WT Arabidopsis to copper sulfate. For seedlings overexpressing cca-miR398, the greening of the cotyledons was greatly reduced when the seeds were germinated on medium containing different supplementary copper sulfate (Figs. 2B–2E). In the 40 mM copper sulfate medium, 84% of the germinated WT seedlings turned green while only 63% of the transgenic seeds turned green after seven days (Fig. 2J). In addition, the transgenic Arabidopsis had significantly shorter (*P* < 0.01) roots than WT plants, regardless of the presence or absence of copper sulfate (Figs. 2F–2I and 2K).

### Effects of copper sulfate on SOD, POD and CAT activity in the leaves of WT and transgenic Arabidopsis

**Figure 3:**
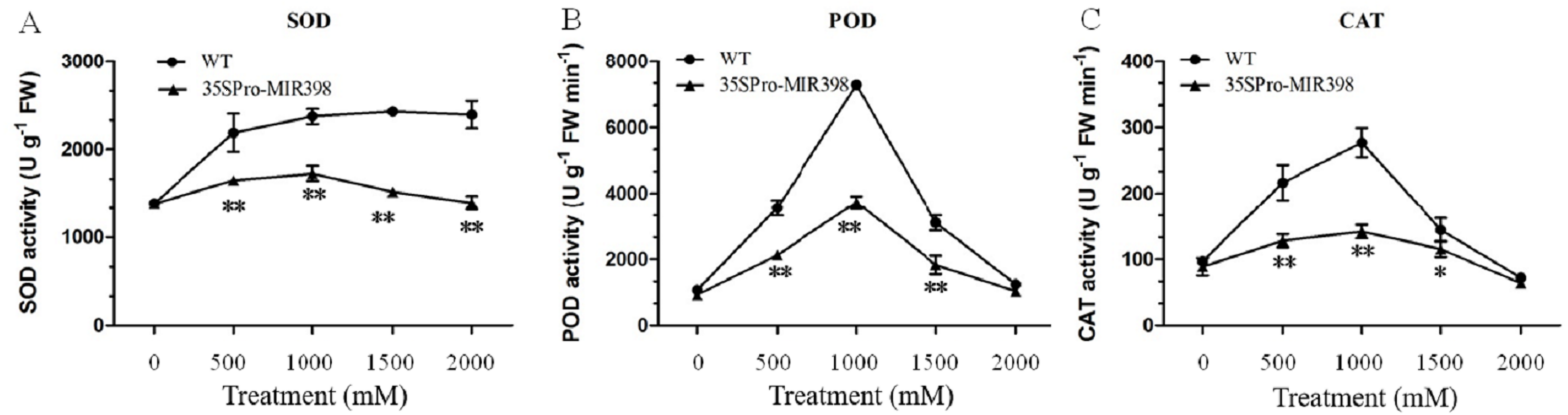
(A) SOD, (B) POD, and (C) CAT activity of Arabidopsis leaves after copper sulfate treatments. Data are means ± SE. Asterisks indicate a significant difference between WT and transgenic lines (*P < 0.05; **P < 0.01).

In general, copper sulfate treatments boosted accumulations of SOD, POD, and CAT more in WT Arabidopsis than in transgenic Arabidopsis. There were almost no differences in the activities between the transgenic and WT lines under normal conditions. As copper sulfate stress increased, the activities of SOD, POD, and CAT increased until 1,000 mM and then decreased (Fig. 3). Under 1,000 mM treatment, SOD, POD, and CAT activities in the transgenic Arabidopsis increased from 1,385, 930, and 89 U g^−1^FW to 1,730, 3,727, and 142 U g^−1^FW, respectively, while SOD, POD, and CAT in the WT plants almost doubled their activities (Fig. 3). There were activity increases for SOD, CAT, and POD at normal conditions to 1,000 mM and a decrease at 1,000 to 2,000 mM stress conditions (figs. 3B and 3C). The transgenic lines showed significantly lower (*P* < 0.01) SOD, POD, and CAT activities than the WT lines after copper sulfate treatments, which may reflect cca-miR398 targeting the three enzymes and thereby reducing antioxidant enzyme activity under stress conditions (Fig. 3).

### Effects of copper sulfate on chlorophyll fluorescence parameters in the leaves of WT and transgenic Arabidopsis

**Figure 4:**
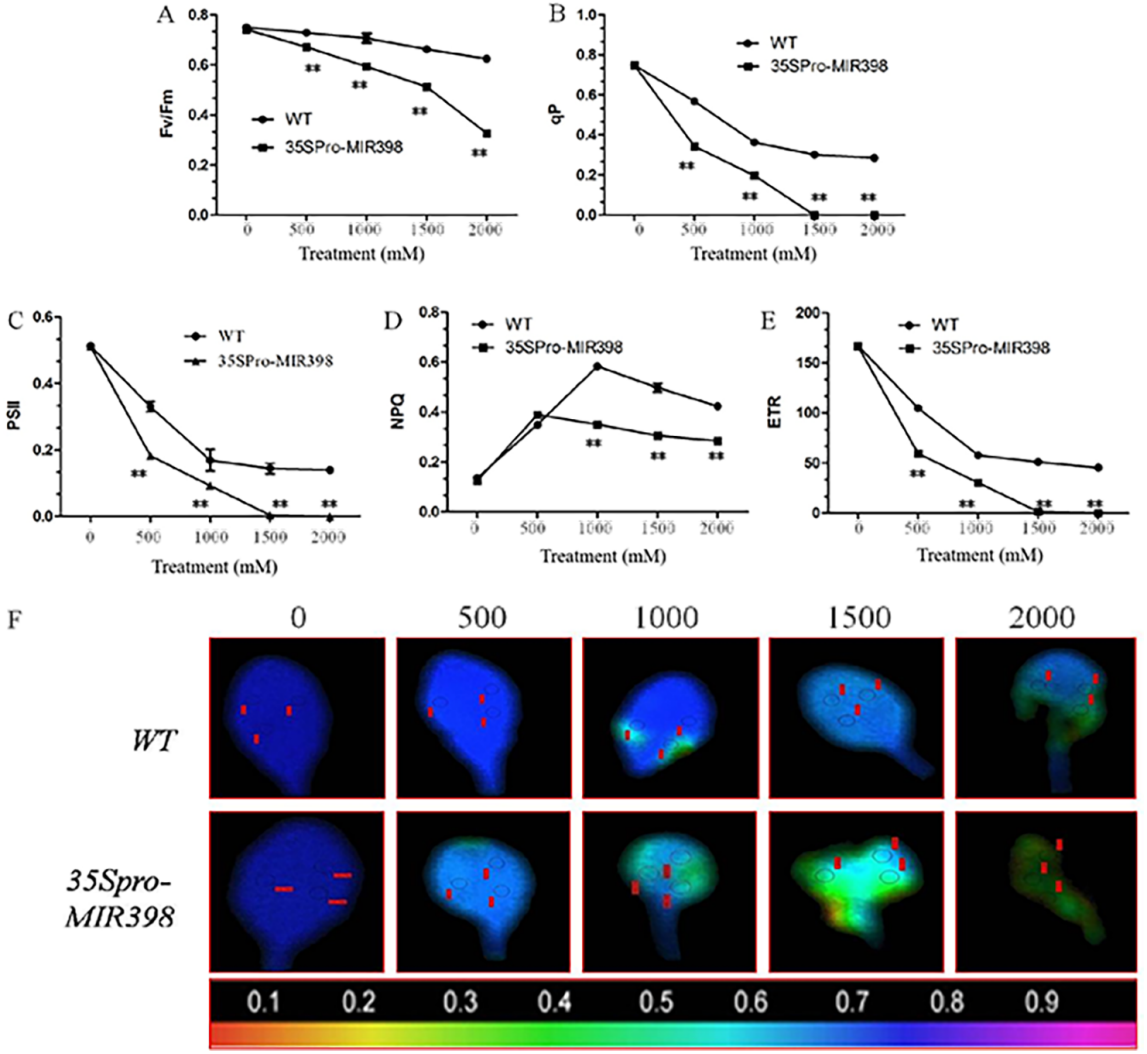
Effects of copper sulfate on chlorophyll fluorescence parameters in the leaves of WT and transgenic Arabidopsis. Effects of copper sulfate on (A) the maximal PSII quantum yield (Fv/Fm), (B) the coefficient of photochemical quenching (qP), (C) effective Y(II) quantum yield, (D) non-photochemical quenching (NPQ), (E) electron transport rate (ETR), and (F) fluorescence images of the maximal Y(II) quantum yield (Fv/Fm) in the leaves of WT and transgenic Arabidopsis. The WT row shows different treatments; the unit is mM, and the number below each row indicates Fv/Fm. Data are means ± SE. Asterisks indicate a significant difference between WT and transgenic lines (**P < 0.01).

The results showed that there were no differences in chlorophyll fluorescence parameters between the transgenic lines and WT under normal conditions. However, the related parameters were significantly lower in the transgenic lines than the WT lines (*P* < 0.01) after copper sulfate treatments. Under copper sulfate treatments, Fv/Fm decreased significantly (*P* < 0.01) in the leaves of transgenic plants when compared to WT leaves (Fig. 4A). Within the range of copper sulfate treatments, qP decreased rapidly between the transgenic lines and WT, reflecting the declining proportion of open PSII reaction centers (Fig. 4B). At a concentration of 1,500 mM copper sulfate or higher, qP began to stabilize, indicating changes in the regulative capability of PSII reaction centers. The capacities of qP in the transgenic Arabidopsis were more sensitive to copper sulfate stress.

Plants in copper sulfate treatments showed that the changing trends of PSII activity were compatible with that of qP (figs. 4B and 4C). Simultaneously, NPQ increased to the highest point under treatments of 500 and 1,000 mM copper sulfate, indicating that WT plants regulated excessive energy dissipation more efficiently than transgenic Arabidopsis (Fig. 4D). The change in ETR curves was similar to that of PSII and the overexpression of cca-miR398 resulted in less ETR susceptibility of ETR to copper sulfate stress (Fig. 4E). In treatments lower than 1,500 mM copper sulfate, ETR in the leaves of transgenic Arabidopsis was almost zero in contrast to the 45.45 ± 1.75µmol m^−2^ s^−1^ in WT leaves.

Images of Fv/Fm in the transgenic Arabidopsis leaves varied with increasing copper sulfate concentrations, which differed from those of WT leaves (Fig. 4F). The imaging color of Fv/Fm in the WT leaves remained partially blue (Fv/Fm = 0.8) with treatments of up to 2,000 mM copper sulfate, although there were small necrotic areas (Fv/Fm = 0). The slow change in the Fv/Fm image color in WT leaves suggested that there was high PSII activity when treated with less than 2,000 mM copper sulfate. The Fv/Fm image colors in the transgenic leaves showed greater degrees of necrosis. The necrosis in the transgenic Arabidopsis leaves accounted for about 90% area of the whole leaf, suggesting that the quantum efficiency of light energy transfer in PSII was almost destroyed in the plants exposed to less than 2,000 mM copper sulfate.

### Copper sulfate stress affecting the expression patterns of cca-miR398, its targets, and copper-responsive miRNAs in WT and transgenic Arabidopsis

**Figure 5:**
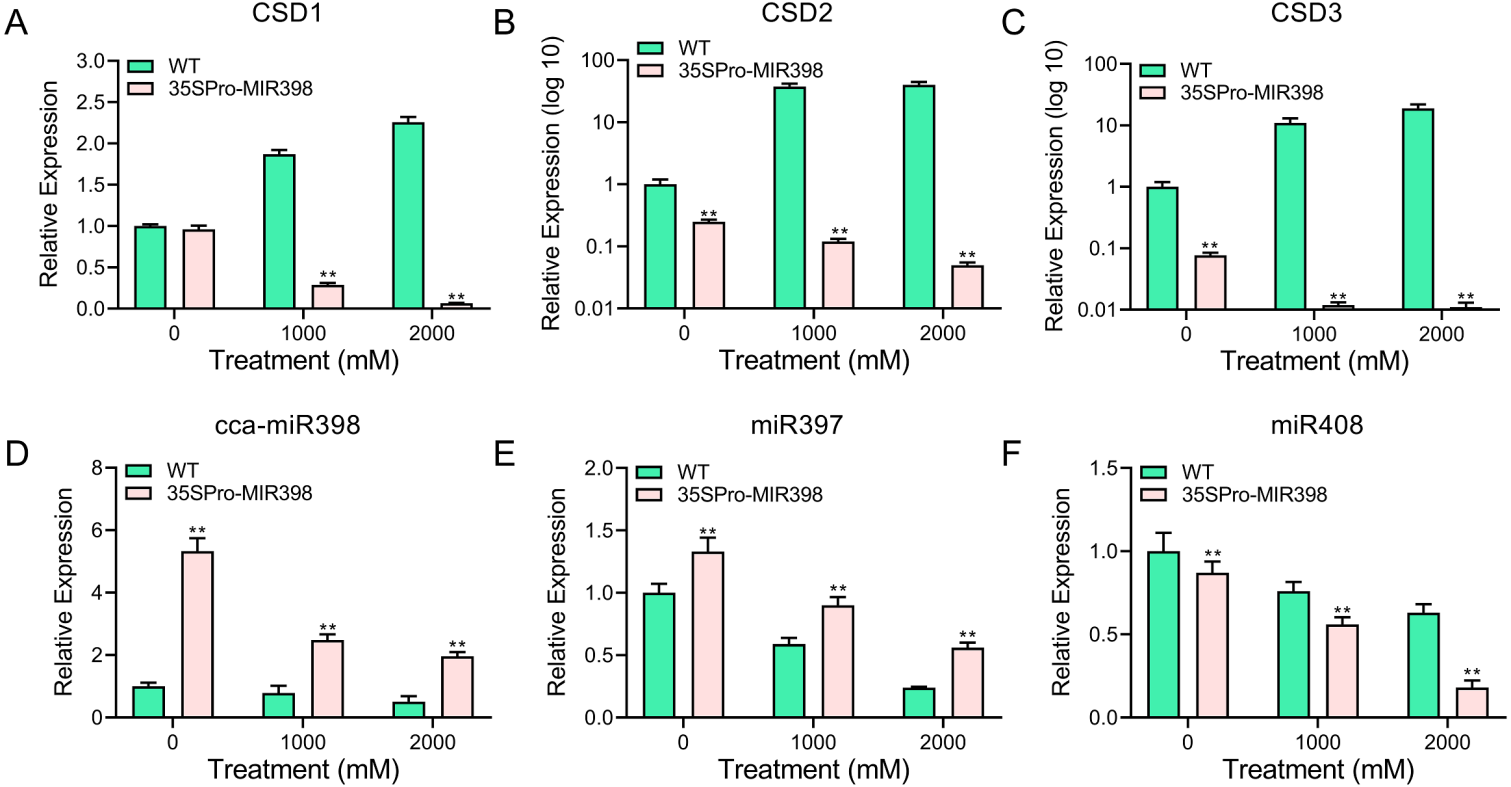
qRT-PCR analysis of cca-miR398, target genes, and copper-responsive miRNAs in WT and transgenic plants subjected to progress copper sulfate stress. The related genes with cca-miR398 or copper sulfate were analyzed in Arabidopsis leaves subjected to 0, 1,000, and 2,000 mM copper sulfate treatments: (A) *CSD1*, (B) *CSD2*, (C) *CSD3*, (D) cca-miR398, (E) miR397, and (F) miR408. Data are means ± SE. Asterisks indicate a significant difference between WT and transgenic lines (**P < 0.01).

qRT-PCR was performed to analyze the transcript abundance of candidate genes in Arabidopsis under copper sulfate stress. The qRT-PCR results showed that the expression of these genes was significantly lower (*P* < 0.01) in the transgenic lines than in the WT lines after copper sulfate treatments (Figs. 5A–5C). *CSD* expression levels increased rapidly with an increase of copper stress in WT plants yet decreased in transgenic plants (Figs. 5A–5C). The results also indicated the involvement of cca-miR398 and *CSDs* in the abiotic stress response of hickory. *CSD3* belongs to *CSD* protein family (Fig. 1), and we could not validate if it was a target of cca-miR398 by 5′-RACE, but we were still able to examine its expression profile based on qRT-PCR. The *CSD3* expression level remained similar to *CSD1* and *CSD2* (Figs. 5A–5C), suggesting that miR398 was involved in the regulation of *CSD3* at the mRNA level.

As expected, cca-miR398 levels increased in all transgenic plants compared with WT Arabidopsis (Fig. 2A), and the expression of cca-miR398 decreased as copper sulfate concentrations increased. We also examined the transcript levels of several copper-responsive microRNAs, including miR397 and miR408, in both WT and transgenic Arabidopsis. The results showed that expressions of miR397 and miR408 were down-regulated under low copper sulfate treatments (Figs. 5E and 5F). The transcript abundance of miR397 was significantly higher (*P* < 0.01) in the transgenic lines compared with the WT lines under different copper sulfate treatments (Fig. 5E); however, the abundance of miR408 was significantly lower (*P* < 0.01; Fig. 5F).

## Discussion

Drought, salinity, and heavy metal post serious threats to plant growth, leading to decline in agricultural production (Jajic, Sarna & Strzalka, 2015). In response to these stresses, plants have developed adaptation mechanisms in which several miRNAs are found to participate. These miRNAs include miR398, which is induced by various stresses in Arabidopsis and tobacco (Chen et al., 2017; Gómez-Arroyo et al., 2018; Lu, Guan & Zhu, 2013). miR398 is also critical in controlling normal plant growth under sucrose, abscisic acid, and copper stress. The functions of cca-miR398 in hickory may be speculated upon based on the conservative properties of miR398 and its target CSDs in model Arabidopsis plants. However, we have provided direct evidence that it is involved in copper sulfate stress tolerance.

The miR398 family is highly conserved among seed plants, which often contain two or three members (Melotto et al., 2014; Waters, McInturf & Stein, 2010) except for eggplant, bean, and rose. In hickory, we identified two miR398 isoforms (miR398a and miR398b) with same sequence. MiR398 had a conserved target (*CSD1*) that has been studied widely (Brousse et al., 2014; Gielen et al., 2016; Naya et al., 2014). We predicted that hickory miR398 would target four *CSD* genes: one *CSD1*, two *CSD2* s, and one *CSD3*. The role of miR398 in copper homeostasis has been described previously for Arabidopsis and other plants (Gielen et al., 2016; Naya et al., 2014). In this study, copper sulfate suppressed expressions of cca-miR398 in hickory roots but induced that of *CSDs*. Root growth showed various inhibitions due to differing copper sulfate stresses, confirming publicly available research on these genes using Arabidopsis under copper sulfate stress (Gielen et al., 2016; Naya et al., 2014). The evidence supports our conclusion that *CSD* regulation by miR398 in hickory is required to allow the hickory to resist the stress.

Furthermore, cca-miR398 over-expressing transgenic plants induced a series of phenotypes, including reduced cotyledon greening and shortened root length, under a treatment of copper sulfate. Plants can activate antioxidative defense systems to protect themselves from the harmful effects of oxidative stress (Guan et al., 2017; Hossain et al., 2015). Several studies analyzed the early production of activated oxygen species in roots following copper excess (Chen et al., 2015; Cuypers, Vangronsveld & Clijsters, 2010), showing a production of excessive reactive oxygen species (ROS) and a stimulation of SOD activity. SODs represent the first line of defense against superoxide accumulation by rapidly converting superoxide to H_2_O_2_ and molecular oxygen (Guan et al., 2017). *CSDs* are arguably the most important SODs, and their roles in plant stress responses are supported by their increased expression under stress and by the phenotypic analysis of a *CSD2* knock down mutant (Guan et al., 2017). *CSD1* and *CSD2* mRNA are induced under copper sulfate stress conditions and our results suggest that copper sulfate stress induced changes in the activities of antioxidative enzymes, including SOD, CAT, and POD. There were increases in SOD, CAT, and POD activities under normal conditions to 1000 mM copper sulfate stress conditions and decreases under 1000 to 2000 mM, suggesting that the ability of Arabidopsis to resist salt stress decreases with increasing copper sulfate stress. We also observed lower SOD activity in cca-miR398 over-expressing plants than in WT plants under similar stress. This result suggests that cca-miR398 is a negative regulator of ROS via SOD activity, conferring plant tolerance to copper sulfate stress.

We performed a comparative analysis of cca-miR398 over-expressing plants to WT plants grown in different copper sulfate stresses to determine if such alteration would significantly reduce the expression levels of miRNA target genes. Our results have shown that overexpression of miR398 in Arabidopsis during copper sulfate stress decreased *CSDs* transcripts, and consequently resulted in a decreasing accumulation of enzymes important for ROS detoxification and oxidative stress tolerance.

## Conclusions

In short, we observed that the growth of the roots of both transgenic Arabidopsis and hickory reduced under copper stress conditions, suggesting that this was due to the function of cca-miR398, which turns to be a negative regulator of copper sulfate tolerance in hickory. Overexpression of cca-miR398 conferred copper sulfate sensitivity and decreased expressions of genes CSD1, CSD2, and CSD3 in transgenic Arabidopsis. The levels of the stress-related physiological indicators were also altered in the transgenic Arabidopsis. Thus, we conclude that cca-miR398 plays a significant role in hickory tolerance to copper sulfate stress.

##  Supplemental Information

10.7717/peerj.9105/supp-1 Table S1 Sequences used in this article Including cca-miR398, Unigene048628, Unigene032278, Unigene032279, Unigene023925, AT1G08830.1, AT2G28190.1, AT5G18100.1, miR397, miR408,

10.7717/peerj.9105/supp-2 Table S2 miR398 sequence information Red font indicates precursor sequence, the blue font indicates mature miR398 sequence.

10.7717/peerj.9105/supp-3 Table S3 List of qRT-PCR primer sequences used for genes

10.7717/peerj.9105/supp-4 Data S4 Raw data: miR398 in hickory

## Additional Information and Declarations

### Data Availability

The following information was supplied regarding data availability: The raw measurements are available in the Supplemental Files. CSD genes of Arabidopsis, poplar, grape, rice, sorghum, and corn are available from Phytozome v 10.3.
